# Validation of Structural Grounds for Anomalous Molecular Mobility in Ionic Liquid Glasses

**DOI:** 10.3390/molecules26195828

**Published:** 2021-09-26

**Authors:** Mikhail Yu. Ivanov, Sergey A. Prikhod’ko, Olga D. Bakulina, Alexey S. Kiryutin, Nicolay Yu. Adonin, Matvey V. Fedin

**Affiliations:** 1 International Tomography Center SB RAS, Institutskaya Street 3a, 630090 Novosibirsk, Russia; o.bakulina@tomo.nsc.ru (O.D.B.); kalex@tomo.nsc.ru (A.S.K.); 2 Boreskov Institute of Catalysis SB RAS, Lavrentiev Avenue 5, 630090 Novosibirsk, Russia; spri@catalysis.ru (S.A.P.); adonin@catalysis.ru (N.Y.A.)

**Keywords:** ionic liquids, glasses, molecular mobility, spin probe, deuteration effect, nanostructure

## Abstract

Ionic liquid (IL) glasses have recently drawn much interest as unusual media with unique physicochemical properties. In particular, anomalous suppression of molecular mobility in imidazolium IL glasses vs. increasing temperature was evidenced by pulse Electron Paramagnetic Resonance (EPR) spectroscopy. Although such behavior has been proven to originate from dynamics of alkyl chains of IL cations, the role of electron spin relaxation induced by surrounding protons still remains unclear. In this work we synthesized two deuterated imidazolium-based ILs to reduce electron–nuclear couplings between radical probe and alkyl chains of IL, and investigated molecular mobility in these glasses. The obtained trends were found closely similar for deuterated and protonated analogs, thus excluding the relaxation-induced artifacts and reliably demonstrating structural grounds of the observed anomalies in heterogeneous IL glasses.

## 1. Introduction

Ionic liquids (ILs) exhibit a number of unusual and advanced properties [1,2,3], making them prospective media for various chemical processes in many fields of modern science and technology [4,5,6,7,8]. ILs are suitable for many applications, including electrolytes in batteries [9,10,11,12], catalyses [5,13], solvents in liquid–liquid extractions [14,15,16], pharmaceutics and medicine [17,18,19,20]; they have also attracted considerable attention as reaction media for many organic reactions [20,21,22,23,24,25,26,27]. The most unusual characteristic of ILs is their nanoscale self-organization, leading to the formation of heterogeneities [28,29]. There are plenty of theoretical investigations regarding the effects of molecular self-organization in bulk ILs [30,31,32,33,34,35,36,37]. It was experimentally proven that charged cationic head groups and anions tend to aggregate in polar nanodomains, and hydrophobic alkyl chains form apolar nanodomains. Experimental approaches such as X-ray diffraction/scattering [38,39,40,41], neutron diffraction/scattering [42,43] and nuclear magnetic resonance (NMR) spectroscopy [44,45,46] have provided great development in the knowledge on heterogeneous micro- and nanostructures of bulk ILs. Recently, extensive studies of heterogeneities in IL glasses were performed using Electron Paramagnetic Resonance (EPR) spectroscopy [47,48,49,50,51,52]. We developed and implemented complex approach including continuous wave (CW) EPR, pulse and time-resolved (TR) EPR [53,54,55,56,57,58,59,60,61]. Since studied ILs are naturally diamagnetic, spin probes (stable radicals or photoinduced triplet states) were used for EPR detection. Variable-temperature CW EPR data show that mobile and immobile local environments coexist within a broad range of temperatures upon transition from glassy to a liquid state of imidazolium ILs [53,56,61]. Pulse EPR allows monitoring small-angle wobbling (stochastic molecular librations) of spin probe that is dictated by a surrounding glassy matrix, as a function of temperature [62]. It has been found that anomalous suppression of molecular mobility is observed near glass transition temperature (*T*_g_) in a series of ILs, typically in a range from ~(*T*_g_ − 60) K to *T*_g_ [56]. Since most media become less dense upon temperature growth, thus favoring more intensive molecular motion, the observed opposite trend was termed structural anomaly. It was assigned to the structural rearrangements occurring in solvation shell of a radical probe, and later research unequivocally demonstrated that alkyl chains are the main cause of these phenomena [61].

**Figure 1:**
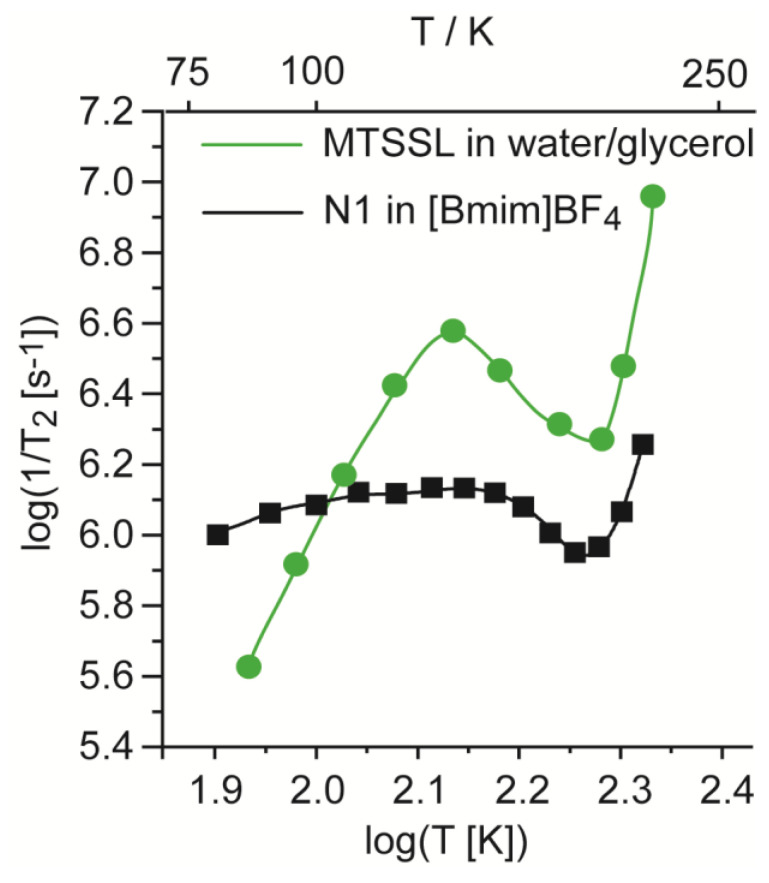
Temperature dependence of spin echo dephasing rates, 1/T_2_, for N1 dissolved in ionic liquid [Bmim]BF_4_ (black squares, adapted from Ref. [56]) and for MTSSL in water/glycerol (1:1) mixture (green circles, adapted from Ref. [63]) measured at the maximum of echo-detected spectrum, position **I** in Figure 2a below.

Still, the detailed mechanism of these structural anomalies has not been revealed. One particular remaining question relates to the role of electron spin relaxation in the observed phenomena. Most anomalies were found to occur at *T*~140–200 K [56]. It is worth noting that approximately the same temperature range is characterized by unfreezing of efficient rotations of CH_3_-groups in various compounds [63,64]. For instance, Figure 1 compares literature data on temperature dependence of transverse relaxation rate (1/*T*_2_) in logarithmic scale for two nitroxide radicals, one of which contains CH_3_-groups adjacent to NO moiety (MTSSL, methane-thiosulfonate spin label, in water/glycerol mixture [63]), and another one does not (N1 radical, see structure below, in IL [56]). Since MTSSL contains methyl groups, these groups have a huge impact on the relaxation behavior in the temperature range where their rotation activates, and the bell-like shape in 1/*T*_2_(*T*) dependence is pronounced. Such temperature range slightly depends on the structure of the radical, but it is present for every nitroxide containing CH_3_ group(s) adjacent to NO moiety [65].

To avoid effects of CH_3_ groups, in our recent studies (including this one) we used N1 radical without methyl groups (see structure below) [63,66,67]. However, elimination of methyl groups from radical might not completely exclude all effects of this kind. Indeed, the cations of IL, which surround radicals, have terminal CH_3_ groups in the vicinity of radical NO group; therefore, this mechanism might still hypothetically contribute to the manifestation of anomaly. This issue is relevant, because in the pulse EPR approach, the molecular mobility in glasses is measured indeed via electron spin relaxation of the embedded probe [62]. Therefore, it is vital to reliably disentangle (i) spin relaxation induced by molecular mobility of the probe (that characterizes local density and structure of heterogeneity) from (ii) spin relaxation induced by rotation of CH_3_-groups in surrounding alkyl chains of IL cations. The relaxation rate of the second process (ii) has a characteristic dependence on temperature with a maximum at *T*~150 K [63], whose shape is generally similar to the dependence of molecular mobility observed by EPR. Several theoretical speculations can be drawn to disentangle two contributions (i) and (ii). The most convincing idea is that relaxation due to mechanism (ii) should be isotropic (i.e., independent of the spectral position) and thus irrelevant for the implemented methodology (vide infra). However, the most transparent experimental verification of mechanisms (i) vs. (ii) might be accomplished by comparing pulse EPR data for protonated and deuterated ILs. The driving force of (ii) is dipolar couplings between the electron of a radical and nuclei of ILs. Since deuteron has a ca. 6.5 times smaller gyromagnetic ratio than the proton, replacement of CH_3_ groups by CD_3_ groups should significantly suppress the magnetic relaxation induced by rotations of these groups [68,69].

The effects of deuteration of a radical’s CH_3_-groups on its phase relaxation have been described in detail in many studies [70,71]. However, the superior way is to replace these methyl groups with cyclohexane groups, which are not able to rotate (N1 radical, see below). Excluding methyl groups of solvent, IL cations, is, of course, not possible, and their deuteration is the only way to distinguish between processes (i) and (ii).

Therefore, in this work we synthesized two deuterated imidazolium-based ILs, [Bmim]BF_4_ and [Bmim]Br, and one pyridinium-based IL, [BuPy]BF_4_, investigated the molecular mobility in their glasses by EPR and compared the obtained results with those for protonated analogs. This experimental study allowed first assessment of the role of methyl groups of IL cations in the observed anomalies.

## 2. Results and Discussion

**Scheme 1:**
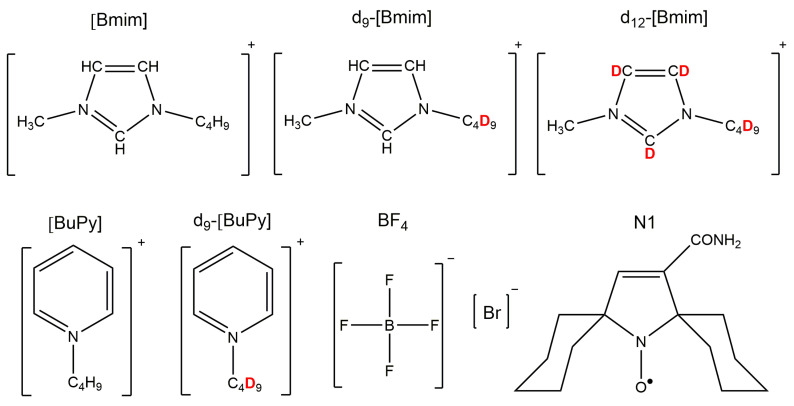
Chemical structures of studied protonated and deuterated ILs and spin probe N1.

Scheme 1 shows the structures of ILs in deuterated and protonated forms studied in this work, along with the structure of nitroxide radical used as a spin probe. We did not succeed in replacing all protons with deuterium in ILs. However, in the case of [Bmim]-like ILs we obtained different isotopomers to check the influence of -CH_3_ groups in imidazolium ring on radical relaxation, as well as to additionally detail the morphology of matrix surrounding the spin probe. Following the same logic, the pyridinium-based IL with non-labile protons in the ring was prepared. Two anions BF_4_^−^ and Br^−^ were selected to exclude relaxation mechanism from rotating fluorine nuclei that have gyromagnetic ratio close to proton [69] and thus might also have an impact on the studied processes.

**Figure 2:**
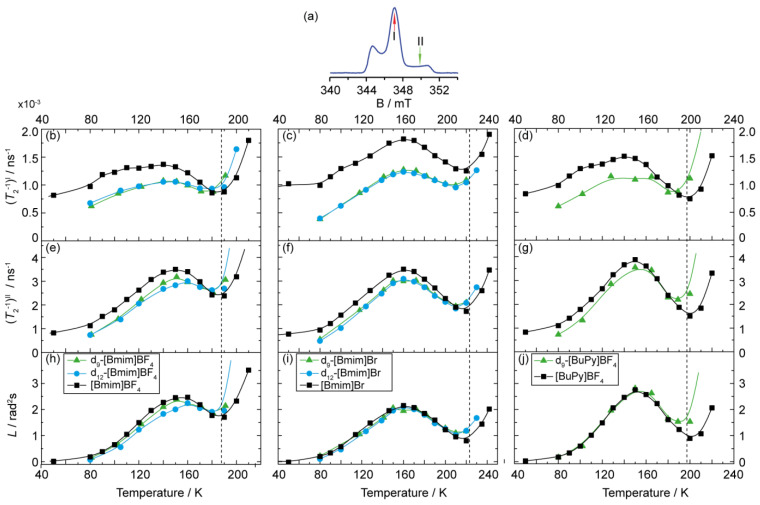
(**a**) Illustrative echo-detected spectrum with indicated field positions **I** and **II** where *T*_2_ was measured, *B* is the magnetic field. (**b**–**g**) Temperature dependence of *T*_2_^−1^ recorded in corresponding spectrum position for protonated and deutered ILs listed in Scheme 1. (**h**–**j**) Temperature dependence of the motional parameter *L* for nitroxide radical N1 in ILs, see text for details. Vertical dashed lines show the corresponding *T*_g_ values for each IL: *T*_g_ = 188 K for [Bmim]BF_4_ [56], *T*_g_ = 223 K for [Bmim]Br [77] and *T*_g_ = 197 K for [BuPy]BF_4_ [56].

To study the molecular mobility in IL glasses, we employed the same approach as that used in our previous works [54,56,61], originally developed by Dzuba and colls [62,72,73] (see Supporting Information for details). Briefly, we investigate small-angle stochastic molecular librations (wobbling) of spin probe located in glass. Several previous studies, which employed radical probes of various structures, have shown that glassy matrix effectively translates its own librational motion onto the embedded probe, thus, the mobility of probe describes the molecular mobility of the glass itself [74,75]. The wobbling of radical modulates magnetic interactions and effectively induces transverse electron spin relaxation with characteristic decay time *T*_2_. For nitroxides at X-band, the main relaxation pathway is caused by electron–nuclear (hyperfine) interactions, whereas the anisotropy of *g*-tensor can be neglected. Due to the anisotropy of the hyperfine interaction between the unpaired electron and ^14^N nucleus, the *T*_2_ depends on field position across the EPR spectrum. The difference of the relaxation rates (1/*T*_2_ values) measured at two characteristic spectral positions (Figure 2a) can be expressed as *L*~(1/*T*_2_^(II)^ − 1/*T*_2_^(I)^) = 10^11^(*α*^2^)*τ*_c_, where (*α*^2^) is the mean-squared amplitude of librations and *τ*_c_ is their characteristic time [56,76]. The temperature dependence *L*(*T*) essentially characterizes the molecular mobility and local rigidity of the matrix surrounding the spin probe.

Figure 2 shows the pulse EPR data for three studied ILs [Bmim]BF_4_, [Bmim]Br and [BuPy]BF_4_ in deuterated and protonated forms (the latter were obtained in our previous research [56]). Figure 2b–g shows the inverse *T*_2_ relaxation time recorded in the spectral positions **I** (b–d) and **II** (e–g), Figure 2h–j reports the *L*(*T*) functions, which are proportional to the difference of inverse *T*_2_ values (see above).

First, it is clear that the anomaly is present in the deuterated glass for all studied ILs. The position of local minimum in *L*(*T*) remains roughly the same and closely coincides with the *T*_g_ temperature of corresponding IL, as is depicted by dashed line. The amplitude of *L*(*T*) in the anomalous region *T*~150–180 K, where molecular mobility decreases with temperature, is slightly smaller in deuterated ILs. However, it is clear that both deuterated and protonated ILs manifest closely the same *L*(*T*), whereas small deviations might arise from the errors of processing the original relaxation data for deuterated IL (see SI for details). It is important to note that the anomaly is also present in 1/*T*_2_ vs. *T* dependences for protonated and deuterated ILs, which means that the temperature-driven librations more strongly contribute to the overall relaxation in this range than the temperature-independent nuclear spin diffusion. Moreover, one can notice that the absolute values of 1/*T*_2_ for deuterated ILs are significantly lower than those in protonated ones. The faster relaxation in the protonated matrix is due to an additional contribution from protons, which by their nature have a larger and more anisotropic magnetic moment than the deuterium nuclei. However, this explanation can be feasible only for comparison between similar ILs but not between different ones, because we cannot exclude the impact of nanostructuring around the spin probe. For example, our previous research with squalene [54], which has a lot of protons, showed slower relaxation compared to some ILs. Importantly, the results for ILs with different deuteration degrees are almost identical. Therefore, protons located in imidazolium ring have a negligible impact on the spin relaxation of radical probe. This indirectly confirms the surrounding of alkyl tails around spin probe, which was earlier proposed in the literature [35,38,44,78], as well as in our previous studies [53,54].

**Figure 3:**
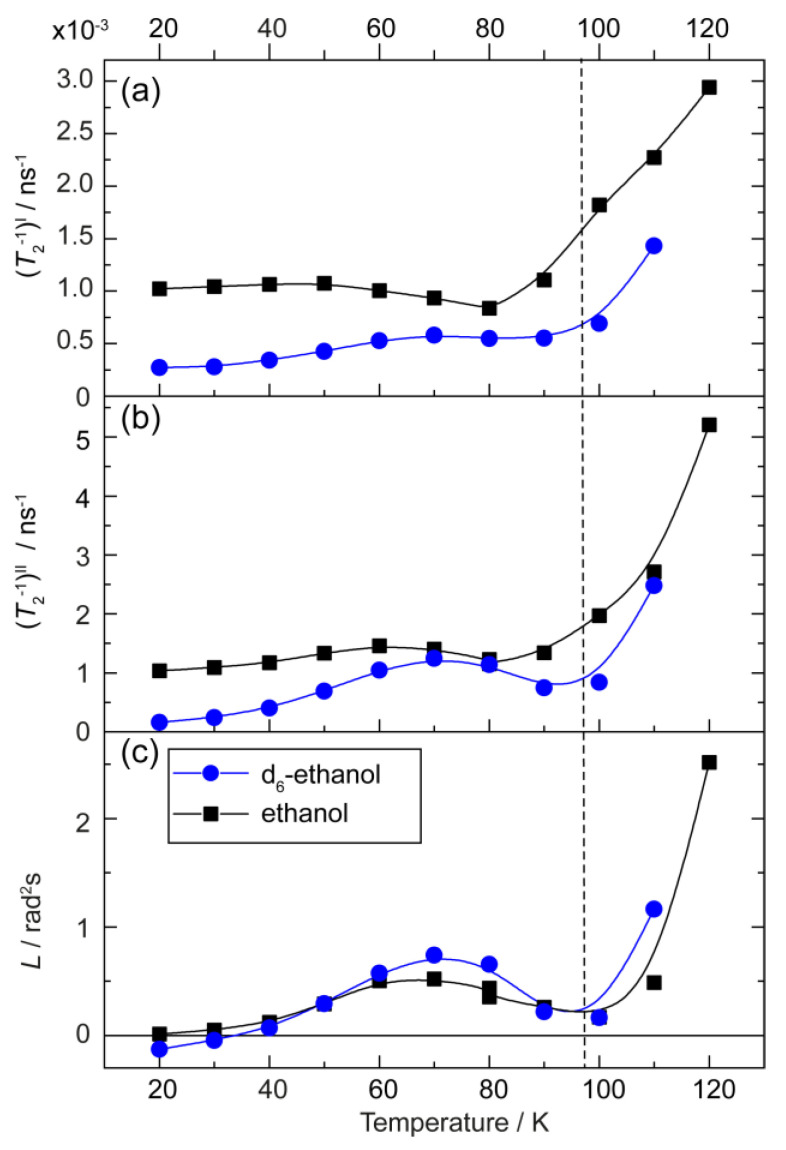
Temperature dependence of *T*_2_^−1^ recorded in spectral positions **I** (**a**) and **II** (**b**) of N1 for protonated and deuterated ethanol. (**c**) Temperature dependence of the motional parameter *L* for nitroxide radical N1 in protonated and deuterated ethanol. The vertical dashed line shows the glass transition temperature for ethanol *T*_g_ = 97 K.

Our recent research has shown that structural anomaly in glassy matrix is governed by alkyl chains of a proper length; in addition, the matrix should have a proper *T*_g_ to allow alkyl chains reveal their dynamical behavior [61]. Recently the anomaly has been observed as well in other compounds apart from ILs, for example in common ethanol. Since fully deuterated ethanol is easily available, we investigated the structural anomaly in this glass as well. Figure 3 shows data for ethanol similar to those reported for ILs in Figure 2. Obviously, similar trends upon deuteration are observed for ethanol and for ILs (see above): the relaxation becomes longer in deuterated glass, but the anomaly *L*(*T*) remains. The d_6_-ethanol glass is completely free of protons, therefore, there is no doubt that deuteration has only minor effects on the shape of the curve.

Thus, we conclude that the anomalous suppression of molecular mobility in ILs near their glass-transition temperatures is a structural phenomenon, not an artifact induced by rotation of methyl groups of solvent activated in approximately the same temperature range. Note that, in principle, the same ‘relaxation-induced artifact’ might originate from rotation of own methyl groups of the radical probe; however, we deliberately used a dedicated spirocyclohexane-substituted nitroxide N1 in this work, which is free from methyl groups adjacent to NO moiety, and because of that has favorable relaxation properties [63,66,67].

The conclusion on structural grounds of the observed anomalies in IL glasses agrees well with that of our previous study, where a different type of spin probe—triarylmethyl (TAM) radical—and different methodology for *L*(*T*) measurement were employed [58]. Despite TAM being much larger that the N1 radical and having drastically different structure and shape, the *L*(*T*) curves were found very similar using both probes, implying that the relaxation (and thus *L*(*T*)) is truly dictated by the librations of glassy environment. Interestingly, in recent works of Dzuba and colleagues, similar *L*(*T*) shapes with a clear maximum were obtained for tumbling radicals attached to the surface (i.e., not in glassy media) [79]. The observation of such saturation near room temperature was explained by violating the applicability of Redfield relaxation theory, when (*α*^2^)*τ*_c_ becomes large due to semi-free large-amplitude motion of the radical. It would be hypothetically possible that in case of glassy ILs radical probes also exhibit similar large-scale tumbling, leading to an anomalous *L*(*T*) shape. However, the coincidence of the *L*(*T*) shapes obtained using nitroxide and TAM radicals requires similar values of their (*α*^2^)*τ*_c_, which seems a very unlikely coincidence for such different molecules. Therefore, with the help of experimental data obtained in the present work, we substantiate structural grounds for the observed *L*(*T*) anomalies in IL glasses.

## 3. Materials and Methods

All deuterated ILs were synthesized following the procedure described in the Supporting Information (SI). We succeeded in obtaining two isotopomers of Bmim cations (d_9_- and d_12_-versions).

The spiro-cyclohexane-substituted nitroxide N1 was kindly provided by Dr Igor Kirilyuk, NIOCh SB RAS. It was dissolved in the corresponding IL in 1 mM concentrations, and the solution was placed in an EPR quartz tube with a 3.8 mm outer diameter.

EPR measurements were performed using a commercial Bruker Elexsys E580 spectrometer at the X-band. The spectrometer was equipped with an Oxford Instruments temperature control system (4–300 K). The echo-detected EPR spectra and phase memory times were recorded using the standard two-pulse echo sequence with pulse lengths being typically 100 ns for π and 50 ns for π/2.

The electron spin echo envelope modulation (ESEEM) was strongly manifested in spin-echo decay of deuterated samples at closely deuteron Larmor frequency (ESEEM for the protonated samples was much weaker for the same pulse lengths used). Therefore, the extraction of corresponding *T*_2_ values in deuterated samples required the fitting procedure accounting for ESEEM, which is described in Figure S1 and text of SI.

## 4. Conclusions

In this work we have provided unequivocal experimental evidence that the observed anomalous molecular mobility near *T*_g_ in imidazolium-based ILs is not a relaxation-induced artifact arising from a rotation of CH_3_-groups in IL cations. Using deuterated ILs and comparing the results with protonated analogs, we safely ruled out the possibility that temperature dependence of transverse relaxation might mimic the suppression of molecular mobility. On the basis of new experimental data and previous pulse/CW EPR studies [61], the observed anomalies should be assigned straight to the structural rearrangements of alkyl chains and increase of local density in apolar domains constituted by these chains. This is an interesting and unique phenomenon, which would be imposed onto any solute molecule localized in such apolar nanodomains. Moreover, recently we have demonstrated that similar structural anomalies are inherent also to certain non-IL solvents, such as phthalates, which are able to form glasses stable in a broad temperature range [61]. Therefore, deeper understanding of such structural anomalies revealed in the present work is of fundamental importance and might also have a variety of implications in the future.

## Data Availability

Not applicable.

## Supplementary Materials

The following are available online: Details on Chemicals. Synthesis of deuterated ionic liquids. The NMR spectral data of ionic liquids. Spin-echo decay simulation.

## Sample Availability

Samples of the compounds are not available from the authors.
