# Supplementary material for: Validation of Structural Grounds for Anomalous Molecular Mobility in Ionic Liquid Glasses
Source: Molecules. 2021 Sep 26;26(19):5828. doi: 10.3390/molecules26195828 (PMC8510339; doi:10.3390/molecules26195828)
Supplement: Supplementary file 1 [file molecules-26-05828-s001.zip › molecules-1366066-supplementary.pdf]

Validation of Structural Grounds for Anomalous Molecular Mobility in Ionic Liquid Glasses

Mikhail Yu. Ivanov,[†,*] *Sergey A. Prikhod'ko,*[§] *Olga D. Bakulina,*[†,‡] *Alexey S. Kiryutin,*[†] *Nicolay Yu. Adonin,*[§] *Matvey V. Fedin*[†,*]

[†]International Tomography Center SB RAS, Institutskaya Street 3a, 630090 Novosibirsk, Russia

[‡]Novosibirsk State University, Pirogova Street 2, 630090 Novosibirsk, Russia

[§]Boreskov Institute of Catalysis SB RAS, Lavrentiev Avenue 5, 630090 Novosibirsk, Russia

Table of Contents

1. Chemicals S1
2. Synthesis of deuterated ionic liquids S1
3. The NMR spectral data of ionic liquids S2
4. Spin-echo decay simulation S4
5. References S5

1. Chemicals

Chlorobutane, N-methyl imidazole, pyridine, sodium tetrafluoroborate and all standard chemicals were obtained from commercial sources. 1-Butanol-d_{10} was purchased from Sigma Aldrich.

The ionic liquids [Bmim]Cl and [BuPy]Cl were obtained by analogy with the literature method[S1] from 1-methyl imidazole or pyridine and chlorobutane. The ionic liquids [Bmim]BF_4 and [BuPy]BF_4 were obtained by previously reported method.[S2]

2. Synthesis of deuterated ionic liquids

D_9-Bromobutane

A 25 ml round-bottomed flask fitted with magnetic stirrer bar and reflux condenser was charged by 6.35 g of 48 mas % HBr solution (37.7 mmol of HBr). 1.85 g (1.05 ml, 19.0 mmol) of 96% H_2SO_4 was slowly added with stirring. After warming to the room temperature, 2.61 g (31.4 mmol) of 1-Butanol-d10 was added with stirring and then 1.61 g (0.92 ml, 17.0 mmol) of 96% H_2SO_4 was added dropwise. The mixture was refluxed for 3 h and then the product was distilled off in a slight vacuum (Bp. about 40°C at 140 torr). The product was washed with saturated $NaHCO_3$ solution and then with water, dried over $MgSO_4$ and distilled. 3.47 g (76%) of d9-brombutane as a colorless liquid was obtained. In the GCMS we observed one peak with molecular ion of mass 146.

[D_9-Bmim]Br and [d_9-BuPy]Br

A 25 ml two-neck round-bottomed flask fitted with magnetic stirrer bar, reflux condenser, dropping funnel and argon supply was charged by solution of 21.5 mmol of N-methylimidazole or pyridine in 5 ml of acetonitrile. The system was filled by argon and heated to 70°C. Then solution of 3.47 g (24.0 mmol) of d_9-bromobutane in 5 ml of acetonitrile was added dropwise. The mixture was refluxed under argon atmosphere for 6 h. The volatile components were distilled off in vacuo. The residue was washed with diethyl ether and dried in high vacuum (10^{-3} bar) at 60°C for 6 hours. The product yield was 55%

[D_{12}-Bmim]Br (by analogy with ref. [S3])

In the glove box a 5 ml round-bottomed flask fitted with magnetic stirrer bar was charged by 2.0 g (8.8 mmol) of d9-BmimBr and catalytic amount of K_2CO_3 (about 3 mg, 0.02 mmol). Outside the glove box 3 ml of D_2O (150 mmol) was added by syringe. The system was supplied by reflux condenser and the mixture was refluxed under argon atmosphere for 3 h. Then water was distilled of in vacuo and the second portion of D_2O (3 ml, 150 mmol) was added. The mixture was refluxed under argon atmosphere for 3 h. Then water was distilled of in vacuo and the residue was dissolved in dry dichloromethane and dried over $MgSO_4$. Then $MgSO_4$ was filtered, solvent was evaporated and product was dried in high vacuum (10^{-3} bar) at 60°C for 6 hours. 1.03 g (55%) of the product as colorless solid was obtained.

BF_4-containing ionic liquids (general procedure)

A 5 ml round-bottomed flask fitted with magnetic stirrer bar and reflux condenser was charged by 5 mmol of corresponding bromide ionic liquid, 0.83 g (7.5 mmol) of $NaBF_4$ and 2 ml of water*. The mixture was stirred for 2 hours. The product was extracted by 3x2 ml of CH_2Cl_2 (organic layer is upper).The solvent was evaporated and then the second portion of of $NaBF_4$ (0.83 g, 7.5 mmol) and 2 ml of water* were added and the resulting mixture was stirred for 3 hours. The product was extracted by 3x2 ml of CH_2Cl_2 (organic layer is upper). The extract was dried over $MgSO_4$ and evaporated in vacuum. The product was dried in high vacuum (10^{-3} bar) at 60°C for 6 hours. Yield of products are 55-65 %. The reaction of sample of ionic liquid with $AgNO_3$ was negative, that demonstrates the absence of Br^- ions in the ionic liquid media.

*For synthesis of [d12-Bmim]BF_4 D_2O was used as solvent.

3. The NMR spectral data of ionic liquids

All NMR spectra were obtained using the Bruker AVANCE III HD spectrometer at 16.7 T and frequencies 700 MHz for protons and 107.5 MHz for deuterons. For each ionic liquid, a set of complementary ^{1}H and ^{2}D NMR spectra were obtained at room temperature. The ^{1}H NMR spectra were obtained for protonated ionic liquids dissolved in deuterated acetonitrile, while for deuterated substances ^{1}H and ^{2}D spectra were obtained in protonated acetonitrile. ^{1}H NMR spectra in protonated solvents were recorded using efficient solvent suppression technique “excitation sculpting” (zgesgp pulse program).[S4] Proton chemical shifts were referenced to residual signal of acetonitrile (δ=1.94 ppm).[S5] Chemical shifts in ^{2}D NMR spectra were adjusted manually for the best correlation with ^{1}H NMR spectra.

1-Butyl-3-methylimidazolium bromide ([Bmim]Br)

^{1}H NMR: δ 9.25 (s, 1H, *H*-2); 7.51 (s, 1H, *H*-4); 7.47 (s, 1H, *H*-5); 4.23 (t, 2H, $^3J_{HH}$ 7.3 Hz, N-CH_2); 3.91 (s, 3H, NCH_3); 1.85 (tt, 2H, $^3J_{HH}$ 7.4 Hz, $^3J_{HH}$ 7.4 Hz, 2-CH_2); 1.35 (qt, 2H, $^3J_{HH}$ 7.5 Hz, $^3J_{HH}$ 7.5 Hz, 3-CH_2); 0.96 (t, 3H, $^3J_{HH}$ 7.4 Hz, 4-CH_3).

1-(d_9-Butyl)-3-methylimidazolium bromide ([d9-Bmim]Br)
^{1}H NMR: δ 9.42 (s, 1H, *H*-2); 7.62 (s, 1H, *H*-4); 7.58 (s, 1H, *H*-5); 3.99 (s, 3H, N-CH_3).
^{2}D NMR: δ 4.25 (s, 2D, N-CD_2); 1.84 (s, 2D, 2-CD_2); 1.33 (s, 2D, 3-CD_2); 0.94 (s, 3D, 4-CD_3).

1-(d_9-Butyl)-3-methyl(2,4,5-deytero)imidazolium bromide ([d12-Bmim]Br)
^{1}H NMR: δ 3.99 (s, 3H, NCH_3).
^{2}D NMR: δ 9.28 (s, 1D, *D*-2); 7.59 (s, 1D, *D*-4); 7.56 (s, 1D, *D*-5); 4.25 (s, 2D, N-CD_2); 1.85 (s, 2D, 2-CD_2); 1.34 (s, 2D, 3-CD_2); 0.96 (s, 3D, 4-CD_3).

1-Butyl-3-methylimidazolium tetrafluoroborate ([Bmim]BF_4)
^{1}H NMR: δ 8.51 (s, 1H, *H*-2); 7.42 (s, 1H, *H*-4); 7.39 (s, 1H, *H*-5); 4.16 (t, 2H, $^3J_{HH}$ 7.3 Hz, N-CH_2); 3.85 (s, 3H, N-CH_3); 1.83 (tt, 2H, $^3J_{HH}$ 7.4 Hz, $^3J_{HH}$ 7.5 Hz, 2-CH_2); 1.34 (qt, 2H, $^3J_{HH}$ 7.5 Hz, $^3J_{HH}$ 7.5 Hz, 3-CH_2); 0.95 (t, 3H, $^3J_{HH}$ 7.4 Hz, 4-CH_3).
^{19}F NMR: δ -152.67 (s, 4F, BF_4).

1-(d_9-Butyl)-3-methylimidazolium tetrafluoroborate ([d9-Bmim]BF_4)
^{1}H NMR: δ 8.54 (s, 1H, *H*-2); 7.46 (s, 1H, *H*-4); 7.43 (s, 1H, *H*-5); 3.90 (s, 3H, N-CH_3).
^{2}H NMR: δ 4.14 (s, 2D, N-CD_2); 1.80 (s, 2D, 2-CD_2); 1.30 (s, 2D, 3-CD_2); 0.92 (s, 3D, 4-CD_3).
^{19}F NMR: δ -151.83 (s, 4F, BF_4).

1-(d_9-Butyl)-3-methyl(2,4,5-deytero)imidazolium tetrafluoroborate ([d12-Bmim]BF_4)
^{1}H NMR: δ 3.91 (s, 3H, N-CH_3).
^{2}H NMR: δ 8.53 (s, 1D, *D*-2); 7.46 (s, 1D, *D*-4); 7.43 (s, 1D, *D*-5); 4.14 (s, 2D, N-CD_2); 1.80 (s, 2D, 2-CD_2); 1.30 (s, 2D, 3-CD_2); 0.92 (s, 3D, 4-CD_3).
^{19}F NMR: δ -151.80 (s, 4F, BF_4).

N-Butylpyridinium tetrafluoroborate ([BuPy]BF_4)
^{1}H NMR: δ 8.75 (d, 2H, $^3J_{HH}$ 5.6 Hz, *o-H*); 8.54 (tt, 1H, $^3J_{HH}$ 7.8 Hz, $^4J_{HH}$ 1.2 Hz, *p-H*); 8.06 (dd, 2H, $^3J_{HH}$ 6.7 Hz, $^3J_{HH}$ 6.7 Hz, *m-H*); 4.56 (t, 2H, $^3J_{HH}$ 7.6 Hz, N-CH_2); 1.97 (tt, 2H, $^3J_{HH}$ 7.4 Hz, $^3J_{HH}$ 7.6 Hz, 2-CH_2); 1.39 (qt, 2H, $^3J_{HH}$ 7.5 Hz, $^3J_{HH}$ 7.5 Hz, 3-CH_2); 0.97 (t, 3H, $^3J_{HH}$ 7.4 Hz, 4-CH_3).
^{19}F NMR: δ -152.40 (s, 4F, BF_4).

N-(d_9-Butyl)pyridinium tetrafluoroborate ([d9-BuPy]BF_4)
^{1}H NMR: δ 8.79 (d, 2H, $^3J_{HH}$ 6.2 Hz, *o-H*); 8.58 (t, 1H, $^3J_{HH}$ 8.0 Hz, *p-H*); 8.11 (bs, 2H, *m-H*).
^{2}H NMR: δ 4.54 (s, 2D, N-CD_2); 1.93 (s, 2D, 2-CD_2); 1.34 (s, 2D, 3-CD_2); 0.93 (s, 3D, 4-CD_3).
^{19}F NMR: δ -152.11 (s, 4F, BF_4).

4. Spin-echo decay simulation

The echo-detected EPR spectra and phase memory times were recorded using the standard two-pulse echo sequence with pulse lengths being typically 100 ns for π and 50 ns for π/2 pulses. The electron spin echo envelope modulations (ESEEM) are present in spin-echo decay, as is illustrated in Figure S1.

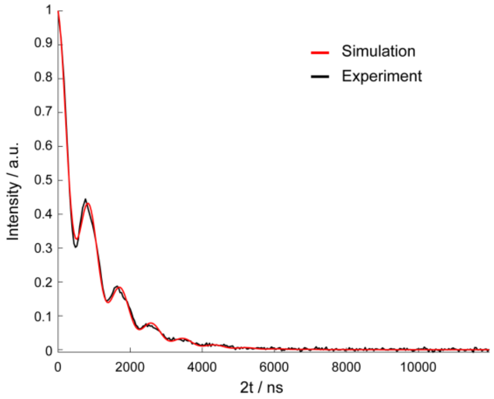

Figure S1. The example of spin-echo decay with ESEEM modulation. Black curve – experimental data, red curve – simulation.

This curve can be described by following expression[S6]

$$V(t) = A[\sin(\omega t + \varphi) + c]e^{-\frac{2t}{T_2}},$$

where ω corresponds to deuterium ESEEM frequency at 9.6 GHz (~2.29 MHz), T_2 – transverse relaxation time, A,c,φ – adjusted constants. As a result, T_2 for corresponding field position at certain temperature had been obtained.

5. References

(S1) Dupont, J.; Consorti, C. S.; Suarez, P. a. Z.; De Souza, R. F. PREPARATION OF 1-BUTYL-3-METHYL IMIDAZOLIUM-BASED ROOM TEMPERATURE IONIC LIQUIDS. *Org. Synth.* **2002**, *79* (September), 236. https://doi.org/10.15227/orgsyn.079.0236.

(S2) Bakulina, O. D.; Ivanov, M. Y.; Prikhod'ko, S. A.; Pylaeva, S.; Zaytseva, I. V; Surovtsev, N. V; Adonin, N. Y.; Fedin, M. V. Nanocage Formation and Structural Anomalies in Imidazolium Ionic Liquid Glasses Governed by Alkyl Chains of Cations. *Nanoscale* **2020**, *12* (38), 19982–19991. https://doi.org/10.1039/D0NR06065H.

(S3) Bottini, G.; Moyna, G. Determining the Relative Strengths of Aromatic and Aliphatic C—H· · · X Hydrogen Bonds in Imidazolium Ionic Liquids through Measurement of H/D Isotope Effects on 19F Nuclear Shielding. *Magn. Reson. Chem.* **2018**, *56* (2), 103–107. https://doi.org/10.1002/mrc.4615.

(S4) Hwang, T. L.; Shaka, A. J. Water Suppression That Works. Excitation Sculpting Using Arbitrary Wave-Forms and Pulsed-Field Gradients. *J. Magn. Reson. Ser. A* **1995**, *112* (2), 275–279. https://doi.org/10.1006/jmra.1995.1047.

(S5) Fulmer, G. R.; Miller, A. J. M.; Sherden, N. H.; Gottlieb, H. E.; Nudelman, A.; Stoltz, B. M.; Bercaw, J. E.; Goldberg, K. I. NMR Chemical Shifts of Trace Impurities: Common Laboratory Solvents, Organics, and Gases in Deuterated Solvents Relevant to the Organometallic Chemist. *Organometallics* **2010**, *29* (9), 2176–2179. https://doi.org/10.1021/om100106e.

(S6) Schweiger, A.; Jeschke, G. *Principles of Pulse Electron Paramagnetic Resonance*, 1st ed.; Oxford University Press: Oxford, 2001.
